# The effects of empagliflozin in patients with type 1 diabetes: Results of a 12-week, double-blind, randomized, placebo-controlled clinical trial

**DOI:** 10.34172/hpp.42486

**Published:** 2024-12-30

**Authors:** Mostafa Najafipour, Farzad Najafipour, Alireza Ostadrahimi, Maryam Ghavami, Zohreh Razaghi, Helda Tutunchi, Naimeh Mesri Alamdari

**Affiliations:** ^1^Department of Internal Medicine, Faculty of Medicine, Azad Ardabil University of Medical Sciences, Ardabil, Iran; ^2^Endocrine Research Center, Tabriz University of Medical Sciences, Tabriz, Iran; ^3^Nutrition Research Center, Department of Clinical Nutrition, Faculty of Nutrition and Food Science, Tabriz University of Medical Sciences, Tabriz, Iran

**Keywords:** Empagliflozin, Sodium-glucose transporter 2 Inhibitors, Type 1 diabetes

## Abstract

**Background:**

Sodium-glucose cotransporter-2 (SGLT-2) acts as a key element in the reabsorption of glucose in the kidney. Currently, SGLT-2 inhibitors are FDA-approved for the treatment of type 2 diabetes. It is suggested that the mechanism of action may operate in the treatment of type 1 diabetes mellitus (T1DM), as well. This study aimed to evaluate the application of empagliflozin as an adjunctive to insulin in patients with T1D.

**Methods:**

In this double-blind placebo-controlled randomized clinical study, 60 type 1 diabetic patients were randomly assigned to have either once-daily empagliflozin 10 mg or placebo, as an addition to insulin for 12 weeks. The hemoglobin A1C, fasting blood sugar (FBS), 2-hour post-prandial blood sugar, and anthropometric indices were measured before and after 12 weeks intervention.

**Results:**

After 12 weeks, empagliflozin resulted in significant reductions of hemoglobin A1C -0.18 (95% CI: -0.37, 0.005, *P*=0.009), FBS –2.60 mg/dL (95% CI: -6.48, 1.28, *P*=0.035), 2-hour post-prandial blood sugar -22.56 mg/dL (95% CI: -35.15, 8.97, *P*<0.0001), and total daily insulin dose –7.6 units (95% CI: -12.4, 2.8, *P*=0.003). Furthermore, empagliflozin reduced body mass index (BMI) by -0.560 kg (95% CI: -0.640, 1.46, *P*<0.0001). Empagliflozin was well tolerated in the patients. Also, no case of hypoglycemia, genital and urinary infections, or diabetic ketoacidosis (DKA) was reported.

**Conclusion:**

The present study supported the use of empagliflozin alongside insulin as a treatment option for individuals with T1D.

**Trial Registration:**

http://www.irct.ir, identifier: irct20130610013612N12, Registration date: 12/9/2022).

## Introduction

 As the fastest-growing chronic disease, type 1 diabetes (T1D) affecting nearly nine million people worldwide.^1^ Since 2000, the prevalence of T1D has risen at four times the rate of global population growth. It is estimated that 17.43 million people will live with T1D in 2040.^2^Additionally, compared to the general population, in individuals with T1D, the risk of cardiovascular diseases is approximately eight times higher, and the risk of mortality from heart disease is even higher. The autoimmune selective deface of pancreatic β-cells, results in a lack of endogenous insulin production and insulin secretion in patients with T1D.^3^

 Multiple daily injections regimens of insulin and frequent monitoring of glucose levels are important components of T1D management.^4^ Although insulin therapy, in comparison with other treatments, leads to a moderately lower all-cause mortality rate, there are still difficulties around T1D treatments, such as weight gain, excessive glucose fluctuations, hypoglycemia, and diabetic ketoacidosis (DKA). The current treatment methods for T1D present a challenge in that a significant number of patients fail to achieve their treatment objectives.^5-7^So, improvement in patient care is needed. One new procedure is adjunctive therapies to insulin treatment.

 Although glycemic status in type 2 diabetes is controlled by different medications, T1D management is mostly confined to insulin therapy.^8,9^ There are several non-insulin medications including, thiazolidinedione, glucagon-like peptide-1 receptor agonists, dipeptidyl peptidase-4 inhibitor metformin, and sulfonylureas that have been assessed in clinical trials, however, they are not currently permitted to be used in patients with T1D.^10^

 A new target organ in the treatment of diabetes is the kidney which may reabsorb glucose more than the normal physiologic renal capacity. The renal glucose reabsorption is regulated by the novel selective inhibitor of the renal proximal tubular “Sodium-glucose cotransporter-2” (SGLT-2), which reduces glucose reabsorption and increases urinary glucose exertion. This can result in low plasma glucose levels in patients with diabetes.^11-13^ The advantage of using SGLT2 inhibitors is the insulin-independent action of these agents. Thus, they may be used as an adjunct to insulin and ameliorate glycemic control in diabetes.^14^ FDA approved dapagliflozin, canagliflozin, ertugliflozin, recently bexagliflozin and empagliflozin (drugs of SGLT2 inhibitors class) for patients with type 2 diabetes. However, there is insufficient clinical evidence to recommend them for T1D.^15,16^

 Animal and human studies explored the beneficial effects of empagliflozin, a potent and selective SGLT2 inhibitor, as an adjunctive-to-insulin therapy in T1D. It can improve glycemia and reduce body weight and hypoglycemia risk.^17-20^ In the phase 3 EASE (Empagliflozin as Adjunctive to Insulin Therapy) study on people with T1D, the dosages of 2.5, 10, and 25 mg of empagliflozin improved glycaemic control, decreased insulin requirements, and lowered weight without increasing the risk of hypoglycemia.^21^ Furthermore, in a systematic review and meta-analysis that evaluated the efficacy and safety of SGLT-2 inhibitors in the treatment of T1D, it was identified that empagliflozin reduced fasting plasma glucose, A1C, body weight, and total daily insulin dose. Moreover, the incidence of adverse events, hypoglycemia, and genital and urinary infections were similar to placebo, while an increased incidence of DKA was detected. The authors suggest that the risk of DKA should be carefully monitored in future clinical trials.^22^ The effect of empagliflozin has not yet been investigated in Iranian populations. Considering that body mass index (BMI), body composition and diet of Iranian populations are different from those in other populations,^23,24^ the present clinical trial was conducted to evaluate the beneficial effects of empagliflozin on HbA1c levels, as a primary outcome, and fasting blood sugar (FBS), blood sugar 2-hour post prandial (BS 2hpp), insulin doses, and anthropometric indices (BMI, waist/hip ratio [WHR], waist/height ratio [WHtR]), as secondary outcomes, in Iranian patients with T1D.

## Methods

###  Study design

 From August 22 to November 21, 2022, a randomized, double-blind, placebo-controlled clinical trial was conducted at Imam Reza Hospital’s endocrinology clinic in Tabriz University of Medical Sciences. The clinical trial protocol was approved by the Ethics Committee of Tabriz University of Medical Sciences, Tabriz, Iran (IR.TBZMED.REC.1401.442). The study was conducted based on the Declaration of Helsinki.^20^The study was registered in the Iranian clinical trial registry (http://www.irct.ir, identifier: irct20130610013612N12, registration date: 12/9/2022). The review boards validated the research procedures and informed consent forms. All participants provided an informed consent form. All methods were carried out based on CONSORT reporting guidelines, and with relevant guidelines and regulations.^25^

###  Sample size

 Using power analysis and sample size software (PASS; NCSS, LLC, US), the sample size was estimated to be 24 for each group. Considering the mean and standard deviation (SD) of FBS (9.0 ± 4.3 and 7.0 ± 3.2 mmol/L) reported by Perkins et al,^18^ the confidence interval (CI) of 95%, the power of 80% in 2-sided tests, and a probable 25% dropout rate, the sample size was increased to 30 patients per group.

###  Inclusion and exclusion criteria

 Key inclusion criteria were adult patients with T1D aged ≥ 18 to ≤ 30 years, and with A1C ≥ 7.5 to ≤ 8.5%. The patients had to be able to measure their blood sugar and daily urine ketone, and also receive multiple daily injections of insulin (glargine/once a day and as part /three time a day), consisting of basal insulin and ≥ 3 daily bolus injections.

 Key exclusion criteria were the use of noninsulin anti-hyperglycemic medicines within three months of inclusion, severe hypoglycemia or DKA, surgery or diet-caused unstable body weight within 3 months of trial, any other experimental medication consumption within 30 days of the study, renal failure and other chronic problems, active infection, urinary tract infection, genital infection, pregnancy, and corticosteroid usage.

###  Treatment and interventions

 Study subjects were randomly assigned in a 1:1 ratio to either the empagliflozin or placebo group through Random Allocation Software (RAS) into one of A (specified as the empagliflozin group) and B (given to the placebo group) treatment groups by the computer-generated allocation schedule and the staff who is not participating in the study. Both patients and investigators were unaware of the randomized therapy allocations. An individual who was not engaged in the research method, assessed patient eligibility and admission, and performed allocation concealment. The assignments were contained inside serially numbered, opaque, sealed envelopes, each designated with a 3-digit code corresponding to the treatments. The intervention group was given empagliflozin (Actoverco Pharmaceutical Company, Iran) at a dose of 10 mg once daily after lunch with their prescribed insulin regimens, and the placebo group was given the same amount of starch tablets synthetized by the drug supplier company (Actoverco Pharmaceutical Company, Iran) which was similar in shape, size, and color with the prescribed insulin regimens for 12 consecutive weeks. Trial medication and placebo were prepared for each group monthly. The intervention’s adherence was assessed during clinic visits. All patients were administered multiple daily insulin injections. They were also instructed to adjust the insulin dosage in accordance with their blood sugar levels and document the daily insulin dose. Diet and exercise counseling was provided to the patients and all were instructed to follow their usual diet, daily carbohydrate intake, and exercise plan as stable as possible all over the study. The patients were provided with self-monitoring of blood glucose devices and urine strips to control urine ketones daily. Hence, in the case of DKA, the patient would have been excluded from the study and stopped using the drug. Participants were followed up to control for any side effects.

###  Anthropometric measurements

 Weight and height were determined with lightweight clothes and no shoes, through a calibrated scale and stadiometer (Seca, Hamburg, Germany) to the nearest 0.1 cm and 0.1 kg, respectively. BMI was computed using the formula: weight (kg)/height (m^2^). Waist circumference (WC), and hip circumference (HC) were measured using a non-stretchable measuring tape to the nearest 0.1 kg. Furthermore, the WHR and WHtR was calculated.

###  Laboratory assays

 At the beginning and the end of the study, 5 cc of blood samples were collected from the participants after 8 to 12-hour of overnight fasting. As soon as the samples were collected in EDTA vacuum blood collection containers (5 mL), they were promptly centrifuged to extract serum. Additionally, the BS was assessed two hours following the typical meal consumption. The enzymatic colorimetric method was employed to determine FBS and BS 2hpp using commercial kits from Pars Azmoon Co, Tehran, Iran. A1C was measured using a commercial kit (BioRex Co., Tehran, Iran) by auto-analyzer (Mindray Auto Hematology Analyzer).

###  Statistical analysis

 Statistical analysis was conducted using IBM SPSS Statistics software (IBM SPSS Statistics, Armonk, USA, version 23). The normality of data distribution was determined using Kolmogorov- Smirnov test. Numerical data were presented as mean (SD) and categorical variables were presented as frequency (percentage), and median (25th, 75th). The Mann-Whitney U test or independent samples t-test was employed to evaluate the differences between the two groups, as applicable. Additionally, the Wilcoxon signed-rank test and the paired samples t-test were implemented to evaluate variations within the groups. Between-and within-group differences of qualitative variables were evaluated using Fisher’s exact test, and Sign test was applied. Analysis of covariance (ANCOVA) test was used to adjust the effects of confounding factors (i.e. baseline values, age, Sex, BMI, and duration of disease).

## Results

**Figure 1:**
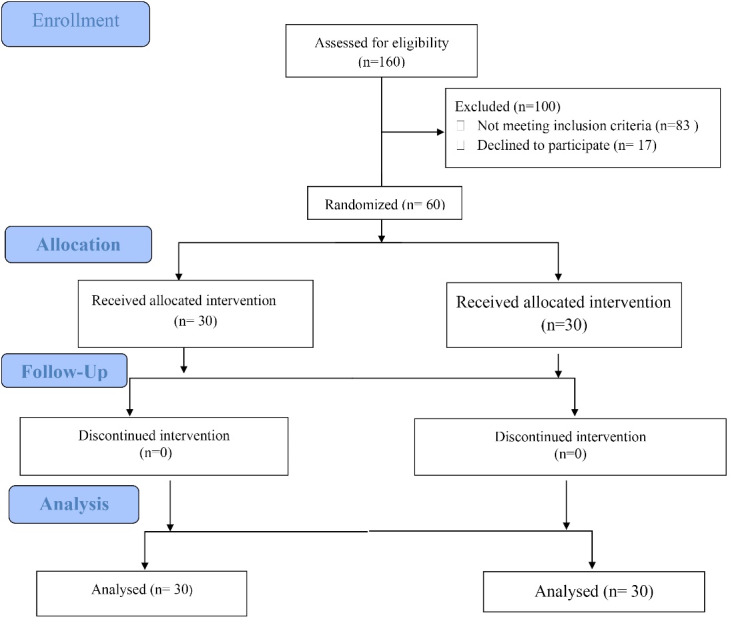
Study flow diagram

 A total of 60 patients were randomized into two groups of placebo (n = 30) and empagliﬂozin

 (n = 30). No patients prematurely discontinued the study. The ﬂowchart of study is shown in Figure 1. The mean age of participants was 25.40 (SD = 9.94) years in the empagliflozin group and 23.13 (SD = 4.50) years in the placebo group. At baseline, there was no statistically significant diﬀerence between the two groups, except for mean weight and BMI which were higher in the empagliﬂozin group (Table 1).

**Figure 2:**
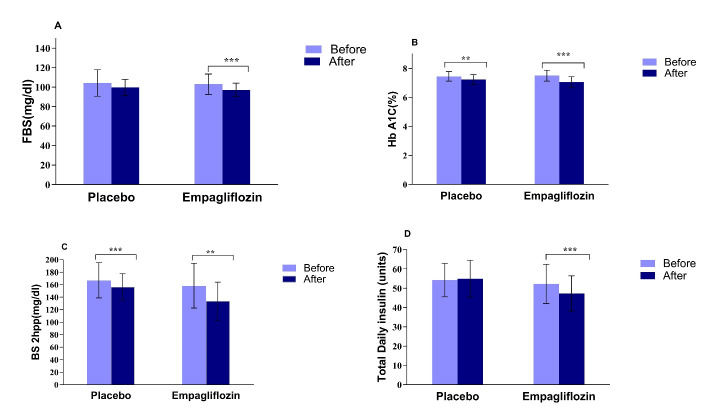
Within group comparison of FBS (A), A1C (B), BS 2hpp (C), and total daily insulin dose (D) before and after intervention. Data are presented as mean (SD). ** *P* < 0.01 by paired *t* test, *** *P* < 0.001by paired *t* test

 The comparisons of mean baseline and after-intervention values of the glucose homeostasis parameters are presented in Figure 2A-2D. A1C and BS 2hpp significantly decreased in both groups after intervention, compared to baseline values (Figure 2B and 2C, *P* < 0.05). Furthermore, FBS (97.20 ± 6.89 mg/dL vs 103.03 ± 10.37 mg/dL, *P* < 0.001) and total daily insulin doses (47.33 ± 9.15 units/day vs 52.27 ± 10.14 units/day, *P* < 0.001) declined significantly at the end of the intervention compared to baseline in the empagliflozin group, but no significant change was found in the placebo group (Figure 2A and 2D).

**Figure 3:**
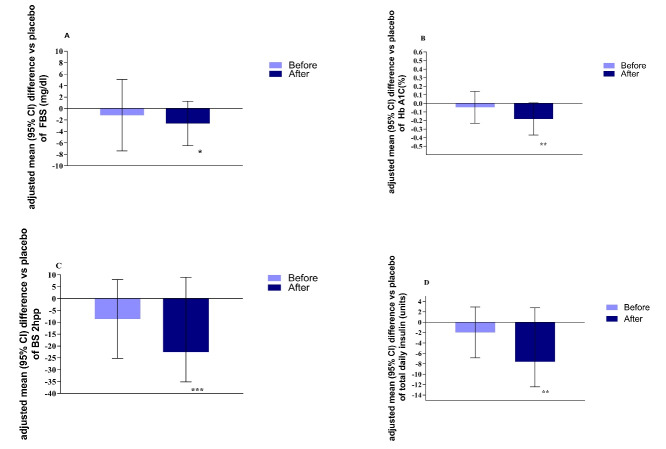
Comparisons of mean placebo-subtracted of FBS (A), A1C (B), BS 2hpp (C), and total daily insulin dose (D) before and after intervention. Data are presented as mean (SD) * P < 0.05 by ANCOVA test (adjusted for baseline values, age, sex, BMI and duration). ** *P* < 0.01 by ANCOVA test (adjusted for baseline values, age, sex, BMI and duration), *** *P* < 0.001 by ANCOVA test (adjusted for baseline values, age, sex, BMI and duration)

 After 12 weeks of treatment, adjusting for the potential confounders (baseline values, age, sex, BMI and duration of disease), empagliflozin resulted in a significant reduction of FBS (-2.06 mg/dL, *P* = 0.035), A1C (-0.18 %, *P* = 0.009,), BS 2hpp (-22.56 mg/dL, *P* > 0.001), and total daily insulin dose (-7.6 units, *P* = 0.003) (Figure 3A-3D).

Table 2 shows that the BMI of participants in the empagliflozin group reduced significantly after the intervention, compared to the baseline (*P* < 0.001). The between-group analysis showed a substantial decrease in BMI values in the empagliflozin group, compared to the placebo group, even after controlling for possible confounders (*P* < 0.001). WHR and WHtR changes were not statistically significant in between-group nor within-group comparisons.

**Table 1:** Baseline characteristic of the study participants

**Variable**	**Placebo (n=30)** **Mean±SD**	**Empagliflozin (n=30)** **Mean±SD**	***P*** ** value **
Age (y)	23.13 ± 4.50	25.40 ± 9.94	0.262^a^
Weight (kg)	63.20 ± 7.97	69.13 ± 6.31	0.002^a^
Height (cm)	164.10 ± 7.06	160.55 ± 5.51	0.137^a^
BMI (kg/m^2^)	23.43 ± 2.25	24.85 ± 1.31	0.004^a^
WC (cm)	75.20 ± 5.03	76.97 ± 6.27	0.234^b^
HC (cm)	82.10 ± 7.94	84.07 ± 6.73	0.051^a^
HbA1C (%)	7.45 ± 0.33	7.5 ± 0.38	0.610^a^
FBS (mg/dL)	104.24 ± 13.558	103.03 ± 10.367	0.24^a^
Duration (y)	4.37 ± 1.92	5.67 ± 3.15	0.061^a^
Insulin daily doses (units/day)	54.23 ± 8.77	52.27 ± 10.14	0.425
Sex, No. (%)			0.592^C^
Female	22 (66.7)	18 (60)	
Male	8 (33.3)	12 (40)	

HC, Hip circumference; BMI, Body mass index; Mean (SD) is presented for data. ^a^ *P* value for independent sample t-test;^b^*P* value for Mann-Whitney U test,^c^* P* value for Fisher’s exact test.

**Table 2:** Anthropometric indices of the study participants throughout the study

	**Placebo (n=30)** **Mean±SD**	**Empagliflozin (n=30)** **Mean±SD**	**MD (95 % CI), ** ***P*** ** value**	***P*** ** value**
BMI			1.42 (0.46,2.37),0.004^b^ -0.560 (-0.649,1.46), 0.002^b^	> 0.001^c^
Baseline	23.43 ± 2.25	24.85 ± 1.31		
Post-treatment	23.33 ± 2.16	23.79 ± 1.22		
MD (95 % CI)	0.36 (0.415, -0.078)	-1. 62(-1.974, -0.808)		
P	0.058^a^	> 0.001^a^		
WHR			-0.025 (-0.068, 0.017), 0.23^g^ -0.039 (-0.081, 0.003), 0.069^g^	0.070^c^
Baseline	0.92 ± 0.09	0.89 ± 0.07		
Post-treatment	0.92 ± 0.09	0.88 ± 0.06		
MD (95 % CI)	0.0001 (-0.004, 0.005)	-0.013 (-0.020, -0.007)		
P	0.938^f^	0.06^a^		
WHtR			0.002 (-0.013,0.198), 0.724^b^ -0.010 (-0.022,0.0108), 0.4^b^	0.505^c^
Baseline	0.45 ± 0.033	0.46 ± 0.031		
Post-treatment	0.45 ± 0.034	0.45 ± 0.030		
MD (95 % CI)	0.010(0.002, 0.038)	0.01 (0.008, 0.013)		
P	0.055^a^	0.058^a^		

BMI, Body mass index; WHR, Waist to hip ratio; Mean (SD) and Mean Difference (95 % CI) are presented for data. ^a^ *P* value for paired *t* test ^b^ *P* value for independent samples *t* test; ^c^ *P* value for ANCOVA test (adjusted for baseline values, age, sex, BMI and duration). ^f^ *P* value for Wilcoxon signed-rank test. ^g^ *P* value for Mann-Whitney U test.

## Discussion

 The prevention of T1D complications needs careful glycemic control. Insulin is the fundamental therapy method in the management of T1D. However, nowadays, there has been a great interest in creating insulin-independent pharmacological therapies to cure patients with T1D.

 SGLT2 inhibitors provide insulin-independent glucose lowering effects by inhibiting glucose reabsorption in the proximal renal tubule, resulting in weight loss and A1C reduction in type 2 diabetes.^26^ However, the therapeutic use of these medications in T1D has not yet been proposed. The present randomized placebo-controlled trial evaluated the eﬀects of empagliflozin as an adjunct therapy to insulin in Iranian participants with T1D. In this trial, empagliflozin 10 mg treatment for 12 weeks improved glycemic control and reduced total daily insulin doses and BMI in patients with T1D. The results of this study were consistent with those of Perkins et al, who evaluated the glycemic efficacy and safety of empagliflozin 25 mg daily in 40 patients with T1D during an 8-week study. They reported the decrement of A1C, FBS, daily insulin dose, hypoglycemic events and weight.^16^ Additionally, in a 4-week placebo-controlled study (EASE-1) among patients with T1D, the administration of empagliflozin for 28 days as an adjunct to insulin increased Urinary Glucose Excretion (UGE), improved A1C, and a reduced weight, significantly. These findings were achieved with lower insulin concentrations than the placebo and without an increase in hypoglycemia.^21,27^ Also, a meta-analysis study showed that the SGLT2 inhibitor treatment, as an adjunct to insulin therapy, remarkably decreased the A1C in comparison with placebo. The reduction rate of A1C ranged from 0.31% to 0.66% relative to the baseline values.^22^ In this study, a 0.5% reduction in A1C was observed with empagliflozin 10 mg, which is a clinically significant finding.

 In our study, no case of DKA was reported, which is considered as an important finding. Our results were inconsistent with the results of Rosenstock et al., who reported that the ketoacidosis rate was increased with 10 mg and 25 mg of empagliflozin, but not with empagliflozin 2.5 mg. They suggested that ketone monitoring leads to early detection and intervention of ketoacidosis and using lower empagliflozin doses may help to reduce this risk.^21^ Moreover, the results of a systematic review and meta-analysis study reported an increased incidence of DKA in SGLT-2 inhibitors group in comparison with the placebo group.^22^ Induction of lower blood glucose levels, which leads to reduced insulin secretion from pancreatic β-cells, and elevation of ketone bodies by activating carnitine palmitoyltransferase-I (CPT-1) or β-oxidation in the liver are the proposed mechanisms of SGLT2 inhibitors that may produce DKA. Moreover, SGLT2 inhibitors promote glucagon secretion, as a result of decrement in insulin secretion, which in turn activates the lipase and fatty acid oxidation leading to ketone bodies production. Finally, in renal tubules, SGLT-2 inhibitors might enhance the absorption of acetoacetate, and prevent the reabsorption of sodium resulting in the increased absorption of the ketone body.^21,27-30^ American Association of Clinical Endocrinologists and American College of Endocrinology advised using the lower SGLT-2 inhibitor doses and not reducing the insulin dose via SGLT-2 inhibitors treatment in future clinical trials.^31^

 The absence of DKA events in the current study may be attributed to the daily ketone monitoring and insulin dose instructions that were implemented during the investigation. Hypoglycemia and weight gain are the primary complications of insulin therapy in T1D, which are dose-dependent.^32,33^ The results of previous clinical trials and those of our study suggested that SGLT-2 inhibitors contribute to the reduction of the total daily insulin dose which seems to be a promising finding.^1,14,21,27^ Based on empagliflozin’s insulin-independent mechanism of action, hypoglycemia events were not observed which is similar to the results of a previous trial by Pieber et al, who evaluated the effectiveness and safety of empagliflozin in patients with T1D during a 4-week study. In their study, in comparison to the placebo, empagliflozin increased urinary glucose excretion, improved A1C, and reduced weight with lower insulin doses over a 28-day period.^14^ Hypoglycemia increased the risk of morbidity and mortality in patients with T1D and has adverse effects on the patients’ quality of life. So, controlling and monitoring hypoglycemia in T1D is essential.^34,35^ Moreover, weight loss was identified with empagliﬂozin as an adjunct to insulin in our study, likely through lowering the insulin dose and calorie loss by stimulating glucose exertion in urine.

 Moreover, empagliﬂozin was well tolerated in the patients, and no event consistent with genital infection was reported.

 Our study was a double-blind, randomized, placebo-controlled trial, among the patient with T1D, which had limitations. A limitation was small sample size which may be the reason for non-significant results in some outcomes. Also, we did not measure the 24-h urinary glucose excretion, which can be an indicative of the Pharmacodynamics of empagliflozin.

## Conclusion

 Empagliflozin, as an SGLT2 inhibitor, acts across insulin-independent mechanisms and is known as an appropriate novel treatment for T1D. Our findings suggest that empagliflozin is a safe and efficacious drug that enhances glycaemic control, reduces body weight, and reduces insulin doses. empagliflozin may aid in the reduction of complications associated with T1D. We also found that empagliflozin does not cause hypoglycemia or genital and urinary infections in T1D. However, future large and long-duration prospective RCTs are suggested to certainly confirm the effects of empagliflozin as an adjunct to insulin in the treatment of T1D.

## Competing Interests

 The authors declare no conflict of interest.

## Ethical Approval

 The experimental protocol was reviewed and approved by the Ethics Committee of Tabriz University of Medical Sciences (IR.TBZMED.REC.1401.442).
